# Presepsin (sCD14-ST) as a new diagnostic biomarker of sepsis: development of diagnostic tools using the whole blood

**DOI:** 10.1186/cc10372

**Published:** 2011-10-27

**Authors:** T Shozushima

**Affiliations:** 1 Department of Critical Care Medicine, Iwate Medical University, School of Medicine, Morika, Japan

## Introduction

CD14 is present in macrophage, monocyte, and granulocyte cells and their cell membranes, and its soluble fraction is present in blood and is thought to be produced in association with infections. It is called the soluble CD14 subtype, and its generic name is presepsin. Presepsin is a novel marker for the diagnosis of sepsis, and the results of previous study in which an ELISA kit was used showed a specific increase in sepsis in the early stage that also correlated well with severity. In the present study we developed a new rapid measurement method for whole blood that use a chemiluminescence enzyme immunoassay. We assessed the usefulness of presepsin values in sepsis.

## Methods

The period of the study was the 10 months from August 2009 to June 2010. The subjects were 41 in-patients, age 62 ± 19 years old, who had been brought to the Critical Care and Emergency Center of Iwate Medical University and who fulfilled at least two of the diagnostic criteria for systemic inflammatory response syndrome (SIRS) on arrival. Blood specimens were collected a total of six times; that is, on admission, and 12 and 24 hours and 3, 5, and 7 days later. Presepsin values were measured by immunoassay analyzer (PATHFAST; Mitsubishi Chemical Medience Corporation, Japan). The sepsis marker PCT, IL-6, and CRP were also measured for comparison. In addition, 128 healthy subjects were assessed as controls.

## Results

The mean presepsin level in the 128 healthy subjects in the control group was 190 pg/ml. The corresponding presepsin levels were normal (non-infection), 294.2 ± 121.4 pg/ml; local infection, 721.0 ± 611.3 pg/ml; SIRS, 333.5 ± 130.6 pg/ml; sepsis, 817.9 ± 572.7 pg/ml; and severe sepsis, 1,992.9 ± 1,509.2 pg/ml; the patients with local infection or sepsis had significantly higher presepsin levels than the patients who did not have infection as a complication. In addition, the presepsin levels in SIRS that was not complicated by infection were significantly lower than in sepsis. When we divided the patients into an infection group and a no infection group and plotted the ROC curves of each of the markers to compare presepsin with other markers, the results showed that presepsin was the best.

## Conclusion

We were able to obtain results similar to those obtained with the conventional ELISA method, and it was possible to diagnose sepsis more rapidly and conveniently using the immunoassay analyzer.

